# Methylseleninic acid induces NAD(P)H:quinone oxidoreductase-1 expression through activation of NF-E2-related factor 2 in Chang liver cells

**DOI:** 10.18632/oncotarget.10289

**Published:** 2016-06-25

**Authors:** Jong-Min Park, Do-Hee Kim, Hye-Kyung Na, Young-Joon Surh

**Affiliations:** ^1^ Tumor Microenvironment Global Core Research Center and Research Institute of Pharmaceutical Sciences, Seoul National University, Seoul, South Korea; ^2^ Department of Food and Nutrition, College of Human Ecology, Sungshin Women's University, Seoul, South Korea; ^3^ Cancer Research Institute, Seoul National University, Seoul, South Korea

**Keywords:** selenium, Nrf2, methylseleninic acid, chemoprevention

## Abstract

Selenium has been reported to induce the expression of some cytoprotective enzymes, which may account for its chemoprotective and chemopreventive effects. However, it remains largely unresolved whether these effects are exerted by selenium itself or mediated by its metabolite(s). In the present study, methylseleninic acid (MSeA), a monomethylated selenium, induced the expression of NAD(P)H:quinone oxidoreductase-1 (NQO-1) in human Chang liver cells. Expression of NQO-1 and other antioxidant/stress response genes is primarily regulated by the transcription factor NF-E2-related factor2 (Nrf2). Exposure of human Chang liver cells to MSeA (3 μM) increased nuclear translocation of Nrf2 and binding to antioxidant response elements. Silencing Nrf2 markedly reduced the MSeA-induced NQO-1 expression. In comparison with embryonic fibroblasts from Nrf2 wild-type mice, those from Nrf2 knockout mice failed to induce NQO-1 expression when treated with MSeA. Moreover, MSeA treatment enhanced ubiquitination of Keap1, but repressed Nrf2 ubiquitination. Pretreatment of cells with dithiothreitol abrogated the MSeA-induced NQO-1 expression, suggesting that MSeA causes Keap1 thiol modification. MSeA-induced NQO-1 upregulation was attenuated in cells harbouring the mutant Keap1 in which the cysteine 151 residue was replaced by serine. Oral administration of MSeA (1 mg/kg) by gavage to mice induced hepatic NQO-1 expression. Similar to MSeA, methylselenol generated from selenomethionine by methioninase activity induced NQO-1 expression. In conclusion, MSeA, the immediate precursor of methylselenol, upregulates the expression of NQO-1 via the Keap1-Nrf2 signaling. The above findings suggest that biological activities of selenium are dependent on the nature of the metabolites as well as the type of ingested selenium formulations.

## INTRODUCTION

Selenium is a trace element essential for human health [1]. Selenium has been shown to prevent tumor formation in numerous animal models and hence to have cancer chemopreventive potential in humans [2, 3]. However, some clinical trials, such as the Selenium and Vitamin E Cancer Prevention Trial (SELECT) and a Phase III trial of selenium, failed to confirm the results of the Nutritional Prevention of Cancer (NPC) trial, implying that the selenium may not be preventive at least against prostate cancer [4, 5]. Such discordant results may be due to the different types of selenium formulations used as well as differential dose responses. Therefore, the biological effects of different forms of selenium and the underlying mechanisms need to be precisely assessed [6-8].

Selenium from different chemical entities include both inorganic selenium and organic forms. Results from *in vitro* studies, animal experiments and clinical trials suggest that the biological activities of selenium are dependent on the type and the nature of metabolites derived from the ingested selenium compound [6, 9]. However, it remains largely unknown which selenium metabolites are more efficacious in cancer prevention, nor are their anticancer and chemopreventive mechanisms fully established.

Methylselenol (CH_3_SeH), a major urinary excretory form of selenium, acts as a precursor for the synthesis of biologically important selenoproteins. Notably, chemopreventive and anticarcinogenic properties of dietary organoselenium compounds have been postulated to result from their metabolic conversion to methylselenol [9, 10]. Methylseleninic acid (CH_3_SeO_2_H; MSeA), a monomethylated selenium compound, has been often used in investigating the biological effects of selenium in cell culture, since it acts as an immediate precursor of methylselenol [11].

NAD(P)H:quinone oxidoreductase-1 (NQO-1), a flavoenzyme that catalyzes two-electron reduction of quinones and its derivatives [12], is a representative phase II carcinogen detoxifying enzyme [13]. Induction of NQO-1 and related antioxidant and carcinogen-detoxifying proteins is known to be regulated by the transcription factor NF-E2-related factor-2 (Nrf2) [12]. Under physiologic conditions, Nrf2 is sequestered in the cytoplasm as an inactive complex with the inhibitory protein Keap1. Keap1 is a cysteine-rich cytoplasmic protein that acts as a redox sensor. Keap1 functions as a substrate adaptor protein for a Cul3-dependent ubiquitin ligase complex and targets Nrf2 for ubiquitination and proteasomal degradation. Dissociation of Nrf2 from Keap1 may represent a key regulatory step that allows this transcription factor to translocate to the nucleus. Nrf2 then binds to stress response elements or antioxidant response elements (ARE) present in the promoter regions of its target genes. One of the most plausible mechanisms responsible for Nrf2 activation involves modification of specific Keap1 cysteine residues. Thus, mutation of Keap1 at Cys-151, Cys-273, and Cys-288 has been shown to impair the ability of Keap1 to repress its transcriptional activity [14].

Here, we report that MSeA induces activation of Nrf2 with concurrent induction of NQO-1 expression, at least in part, via cysteine thiol modification of Keap1, which may account for its chemopreventive and cytoprotective effects.

## RESULTS

### MSeA induces NQO-1 expression in human Chang liver cells

In an initial experiment, we first compared the ability of selected selenium metabolites to induce expression of the NQO-1 in human Chang liver cells. After treatment of cells with each of the selenium metabolites, MSeA (precursor of methylselenol), sodium selenite (precursor of hydrogen selenide), and dimethyl selenide, NQO-1 expression was measured by Western blot analysis. As shown in Supplementary Figure 1, MSeA (1, 2, and 3 μM) significantly induced NQO-1 expression in a concentration-dependent manner. Under the same experimental conditions, the other two compounds did not significantly increase NQO-1 expression. Based on these observations, we attempted to explore the mechanisms by which MSeA induces NQO-1 in the same cell line.

### MSeA induces NQO-1 expression and elevates NQO-1 activity

**Figure 1:**
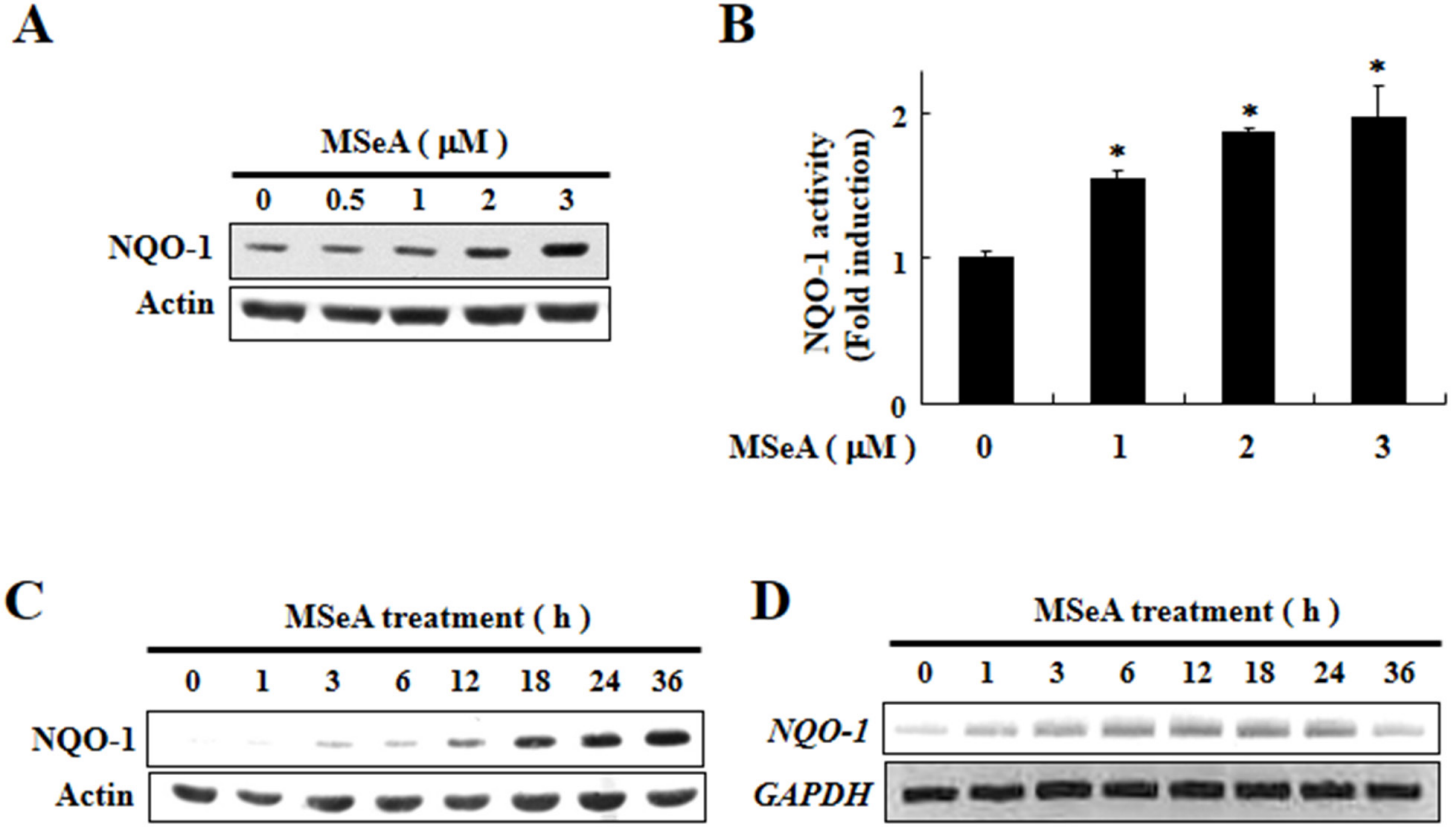
Induction of expression and activity of NQO-1by MSeA **A.** Chang liver cells were incubated with v MSeA (0, 0.5, 1, 2, and 3 μM) for 24 h and harvested for measuring NQO-1 expression by Western blot analysis. **B.** Chang liver cells were treated with MSeA (0, 1, 2, and 3 μM) for 24 h and NQO-1 activity was determined as described in Materials and Methods. **C.** Chang liver cells were treated with MSeA (3 μM) for the indicated durations. The same membranes were probed again with the antibody against actin to ensure equal loading of cellular proteins on the gel. **D.** Chang liver cells exposed to 3 μM MSeA were harvested at the indicated times, and total RNA was prepared. The RNA samples were analyzed by RT-PCR for measuring NQO-1 mRNA expression. GAPDH was used as a control for equal loading. The data represent mean ± SD (n = 3). Significant differences between the compared groups are indicated (*P < 0.05).

To determine the optimal dose range for MSeA, Chang liver cells were treated with varying concentrations of MSeA, and the cell viability was determined by the diphenyltetrazolium bromide (MTT) assay. MSeA, up to a concentration of 3 μM, did not elicit any toxic effects on cell viability for 24 h (Supplementary Figure 2). Thus, non-cytotoxic concentrations of MSeA (1-3 μM) were used for subsequent experiments. Treatment of Chang liver cells with MSeA resulted in concentration-dependent increases in the expression (Figure 1A) and activity (Figure 1B) of NQO-1. MSeA at 3 μM stimulated expression of NQO-1 (Figure 1C) and its mRNA transcript (Figure 1D) in a time-dependent manner.

### MSeA increases nuclear translocation of Nrf2 and its binding to the consensus ARE sequence of NQO-1 gene promoter

**Figure 2:**
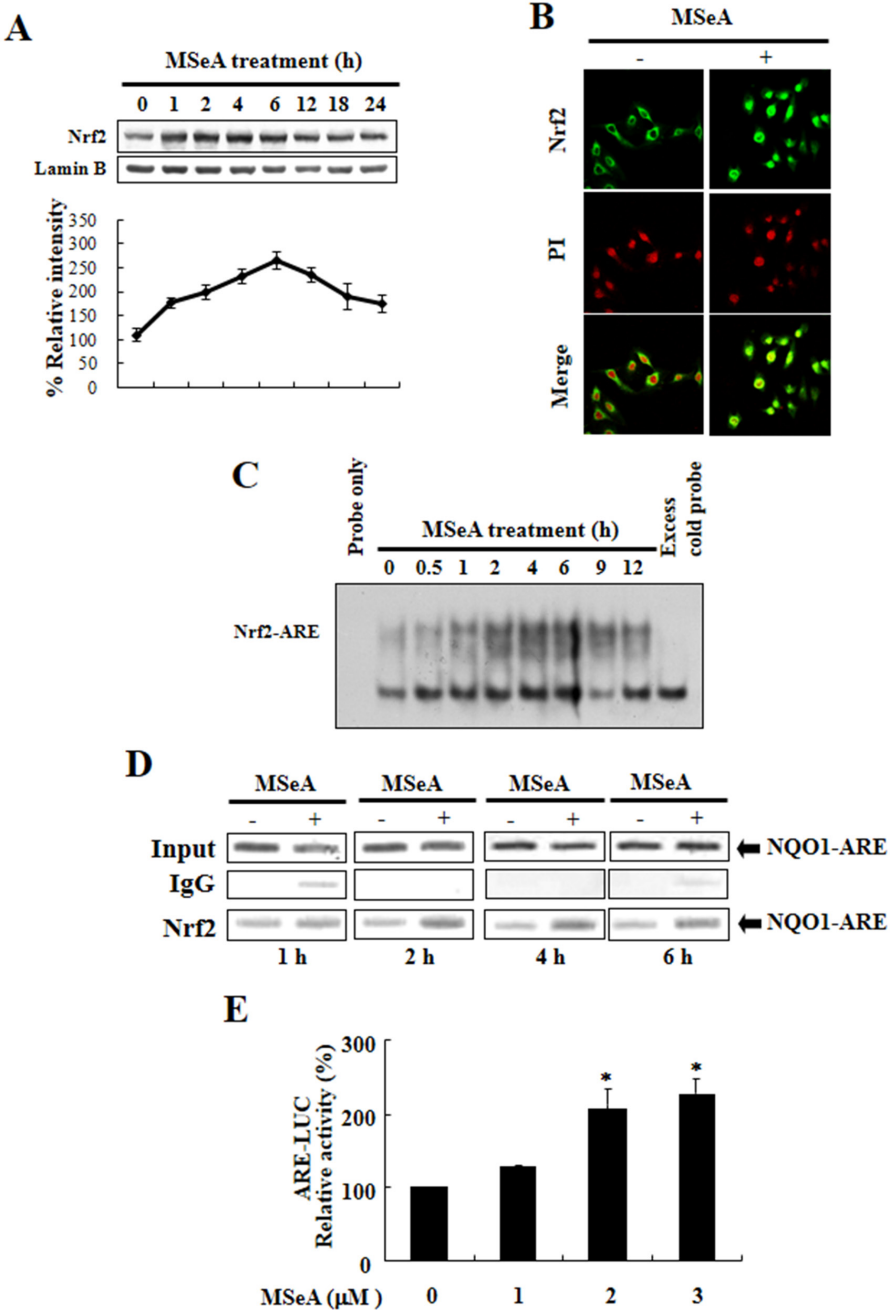
MSeA-induced nuclear translocation, ARE binding, and transcriptional activity of Nrf2 **A.** Effects of MSeA on the levels of nuclear Nrf2. Nuclear extract from Chang liver cells was prepared at the indicated intervals after treatment with 3 μM MSeA. Immunoblots of nuclear lysates were probed with the Nrf2 antibody. **B.** Immunocytochemical analysis of Nrf2 translocation. Chang liver cells were treated with 3 μM MSeA for 6 h. The immunofluoresence staining of Nrf2 was conducted. Propodium iodide (PI) was used as a nuclear counter stain. **C.** Time course of MSeA (3 μM)-induced activation of Nrf2-ARE binding. The nuclear extract isolated from MSeA-treated cells was used for EMSA. The competition assay for the Nrf2-ARE binding was carried out in the presence of 100-fold molar excess of unlabeled oligonucleotide. **D.** The ChIP assay was performed to examine the interaction of Nrf2 with ARE in Chang liver cells treated with 3 μM MSeA for the indicated durations. The cross-linked chromatin was immunoprecipitated with rabbit IgG (control) and anti-Nrf2. Immunoprecipitated chromosomal DNA was analyzed by PCR with primers specific for the human NQO-1 gene ARE. **E.** The MSeA-mediated transcriptional activation of ARE was measured by the luciferase reporter assay (*P < 0.05).

As Nrf2 plays an essential role in anti-oxidant gene expression, we examined the nuclear translocation and ARE binding of Nrf2 in cells treated with MSeA. As shown in Figure 2A, the proportion of Nrf2 translocated into nucleus increased transiently after the MSeA treatment. The nuclear translocation of Nrf2 in MSeA treated cells was further confirmed by the immunocytochemical assay (Figure 2B). Moreover, electrophorectic mobility gel shift assay (EMSA) results revealed that MSeA enhanced the formation of Nrf2-ARE binding complexes (Figure 2C). The specificity of the MSeA-induced Nrf2-ARE binding was verified by a competition experiment in which addition of an 100-fold molar excess of the unlabeled human ARE oligonucleotides completely blocked visible formation of the radio-labled ARE-Nrf2 protein complex (Figure 2C last lane). The Nrf2 binding to ARE was more precisely determined by a chromatin immunoprecipitation (ChIP) assay. As illustrated in Figure 2D, MSeA enhanced the binding of Nrf2 to the ARE. The increased Nrf2-ARE binding was accompanied by enhanced Nrf2 transactivation activity, as assessed by the luciferase reporter gene assay (Figure 2E). Combined, these results suggest that MSeA induces NQO-1 expression in Chang liver cells by activation of Nrf2 signaling.

### MSeA-induced NQO-1 upregulation is mediated via Nrf2 signaling

**Figure 3:**
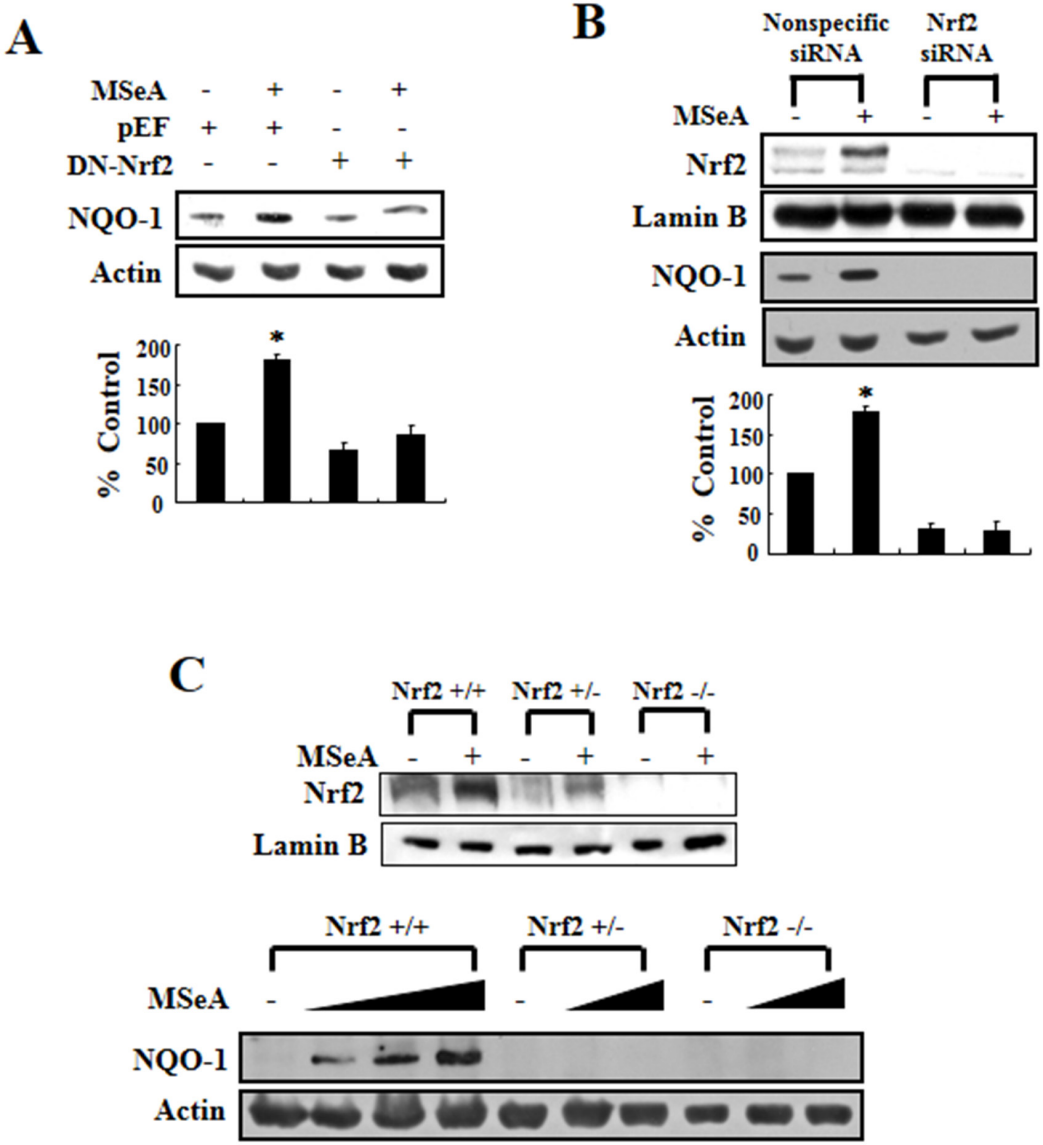
Nrf2 dependence of NQO-1 induction **A.** Chang liver cells were transfected with pEF (blank vector) or dominant-negative mutant Nrf2 (DN-Nrf2) construct using lipofectamine 2000 transfection reagent. After 24-h transfection, cells were treated with 3 μM MSeA and incubated for an additional 24 h. Protein from cell lysates was subjected to Western blot analysis to measure the NQO-1 expression. **B.** Chang liver cells were transfected with nonspecific or Nrf2 siRNA using lipofectamine^®^ RNAiMAX transfection reagent. After 24-h transfection, cells were treated with 3 μM MSeA for an additional 24 h. Protein extracts from cell lysates were analyzed by Western blot with NQO-1 and Nrf2 antibodies (*P<0.05). **C.** Embryonic fibroblast of wild type (*Nrf2*+/+) or knock-out (*Nrf2*+/-, *Nrf2*-/-) mice were incubated with varying concentrations of MSeA (0, 1, 2, or 3 μM) for 24 h and harvested for Western blot analysis to measure expression of NQO-1 and Nrf2.

To further confirm that the expression of NQO-1 induced by MSeA was due specifically to Nrf2-mediated transcriptional regulation, we conducted additional experiments using Chang liver cells transfected with the dominant-negative mutant of Nrf2. Compared with the cells transfected with a vector harboring a functionally active intact Nrf2, cells transfected with the plasmid carrying a dominant-negative mutant form of Nrf2 with truncated N-terminal sequences barely expressed NQO-1 upon MSeA treatment (Figure 3A). Likewise, the MSeA-induced upregulation of NQO-1 in Chang liver cells was abolished by silencing of Nrf2 gene expression with specific siRNA (Figure 3B). Finally, we utilized mouse embryonic fibroblasts (MEFs) obtained from Nrf2 knockout mice to confirm that the upregulation of NQO-1 expression was specifically induced by Nrf2. While prominent induction of NQO-1 expression occurred in the Nrf2 wild-type MEF cells treated with MSeA, MEFs from the Nrf2 knockout (*nrf2*^*-/-*^) mice failed to upregulate NQO-1 expression when treated with 1 or 3 μM of MSeA (Figure 3C). Although Nrf2 is still detectable in the liver of heterozygous (*nrf2*^*+/-*^) mice, this amount of Nrf2 does not appear to be sufficient to induce expression of its target protein NQO-1 (Figure 3C).

### MSeA reduces the Keap1 protein level

**Figure 4:**
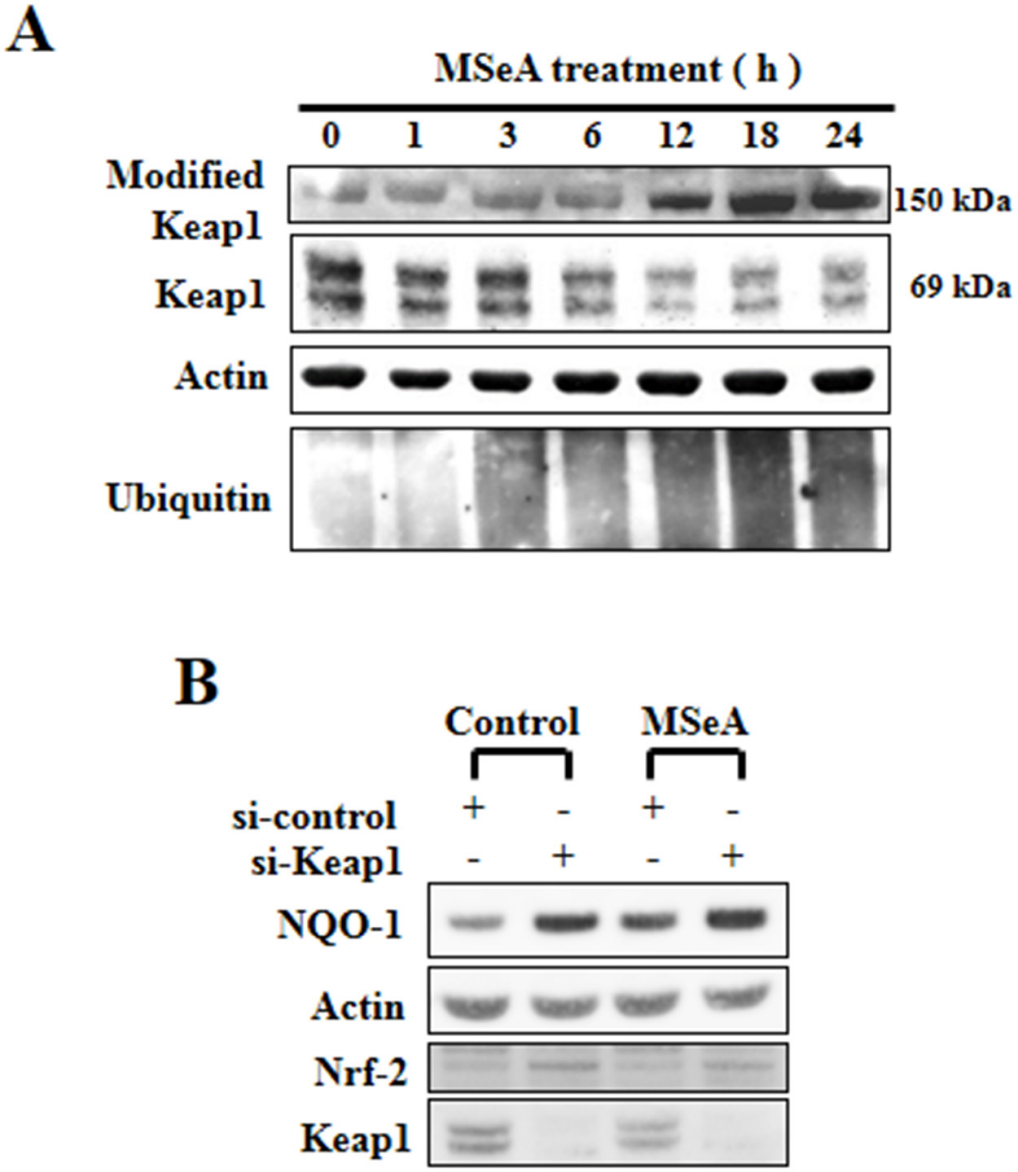
Effects of MSeA on the steady-state levels of Keap1 **A.** MSeA decreased the ratio of Nrf2/Keap1 and increased accumulation of high molecular weight Keap1 forms. Chang liver cells were treated with 3 μM MSeA for the indicated times followed by Western blot analysis with Keap1 and ubiquitin antibodies. **B.** Chang liver cells were transfected with nonspecific or Keap1 siRNA using lipofectamine^®^ RNAiMAX transfection reagent. After 24-h transfection, cells were treated with 3 μM MSeA for an additional 24 h. Protein extracts from cell lysates were analyzed by Western blot with NQO-1 and Nrf2 antibodies.

Under unstimulated conditions, Nrf2 is sequestered in the cytoplasm as an inactive complex with Keap1 [15]. However, disruption of the Keap1-Nrf2 complex allows Nrf2 to translocate into the nucleus and bind to the ARE [16]. Notably, the steady-state level of Keap1 was gradually decreased upon MSeA treatment with concomitant accumulation of the high molecular weight (150 kDa) form of Keap1 (Figure 4A), indicative of Keap1 modification. To confirm whether the decreased level of Keap1 is essential for the MSeA-induced increase of Nrf2-ARE activity, we transfected the Chang liver cells with Keap1 siRNA. As shown in Figure 4B, the basal level of Nrf2 accumulated in the nucleus and constitutive expression of its target protein NQO-1 were increased by silencing Keap1. However, there was no further increase in levels both Nrf2 and NQO-1 in Keap1 knockdown cells when exposed to MSeA, indicating that MSeA interplays with Keap1 in activating Nrf2 and subsequently inducing target gene expression (Figure 4B).

### MSeA enhances ubiquitination of Keap1, but inhibits ubiquitination of Nrf2

**Figure 5:**
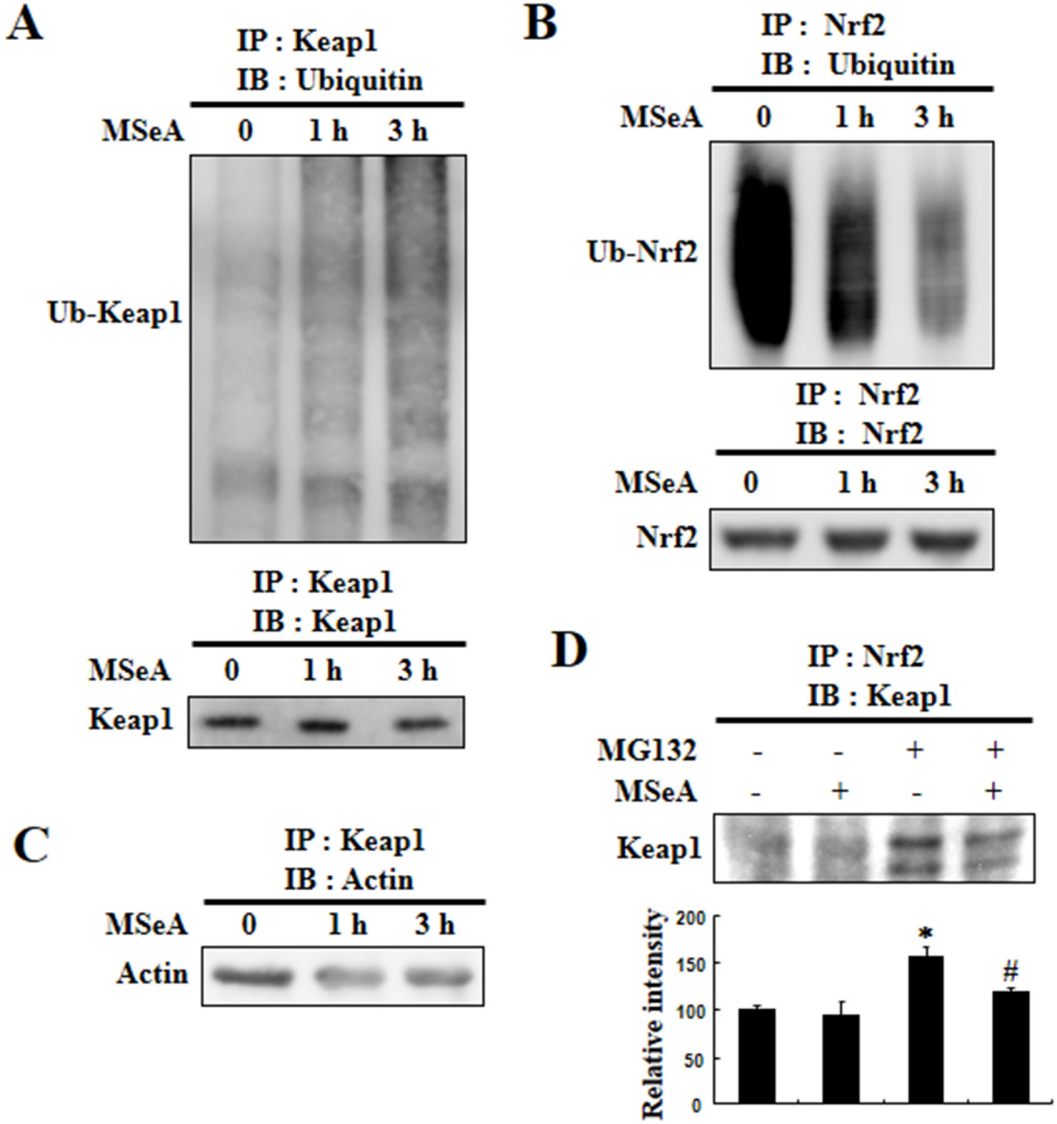
Effects of MSeA on ubiquitination of Nrf2 and Keap1 **A, B.** Chang liver cells were treated with or without 3 μM MSeA for 3 h. Equivalent amounts of proteins were immunoprecipitated with anti-Keap1 antibody (A) or anti-Nrf2 antibody (B) and subjected to Western blot analysis with ubiquitin antibody. **C.** Equivalent amounts of proteins were immunoprecipitated with anti-Keap1 antibody followed by Western blot analysis with actin antibody. **D.** Chang liver cells were treated with 10 μM MG132 for 2 h and then treated with or without 3 μM MSeA for 6 h. Equivalent amounts of proteins were immunoprecipitated with anti-Nrf2 antibody and visualized by Western blot analysis with Keap1 antibody (*P < 0.01 vs. Control, #*P* < 0.05 vs. MG132 ).

It has been reported that Nrf2 is rapidly degraded through the ubiquitin-dependent proteasome pathway under homeostatic conditions [14]. Keap1 functions as a substrate adaptor protein for Cul3-dependent ubiquitin ligase complex and directs degradation of Nrf2 by facilitating ubiquitination and proteasomal degradation. Likewise, Keap1 is also subjected to ubiquitin-proteasomal degradation. To determine whether a decrease in Keap1 and a subsequent increase in Nrf2 in cells treated with MSeA were due to enhancement of Keap1 ubiquitination and degradation and/or to suppression of Nrf2 ubiquitination and degradation, we examined the levels of ubiquitinated Nrf2 and Keap1 by immunoprecipitation. There was a marked increase in the level of ubiquitinated Keap1 (Figure 5A) with a concomitant decrease in the level of ubiquitinated Nrf2 in cells treated with MSeA (Figure 5B). These results suggest that the MSeA-enhanced nuclear translocation of Nrf2 is attributable, at least in part, to the inhibitory effect of MSeA on the ubiquitination and degradation of Nrf2. Under the physiological conditions, Keap1 is anchored to the actin protein in the cytoplasm. MSeA treatment caused decreased association of the Keap1-actin complex (Figure 5C). We speculate that MSeA, through direct interaction with Keap1, stimulates the proteasomal degradation of Keap1, limiting its association with actin. Alternatively, modification of the Keap1 structure by MSeA will diminish the association of Keap1 with Nrf2. This will facilitate the liberation of Nrf2 from Keap1. To test this possibility, we performed a co-immunoprecipitation experiment with anti-Nrf2 and anti-Keap1 antibodies. MSeA treatment lowered the Keap1-Nrf2 complex formation which was visible when Nrf2 degradation was blocked by the proteasomal inhibitor MG132 (Figure 5D).

### MSeA enhances NQO-1 expression through thiol modification of Keap1

Mild oxidative stress and activation of phosphoinositide 3-kinase (PI3K)/Akt caused by some electrophiles have been reported to induce nuclear localization of Nrf2 and subsequent induction of NQO-1 [17]. To explore the signaling events leading to NQO-1 expression in Chang liver cells treated with MSeA, we used several modulators of intracellular reactive oxygen species (ROS) generation and inhibitors of PI3K/Akt activation. These include reduced glutathione (GSH), the GSH depletor buthioninesulfoximine (BSO), the GSH precursor *N*-acetyl-L-cysteine (NAC), the ROS scavenger vitamin C, and the PI3K/Akt signaling inhibitors LY294002, Wortmanin, or the Akt inhibitor II. None of these compounds had significant inhibitory effects on MSeA-induced NQO-1 expression (Supplementary Figure 3A-3D). These findings suggest that ROS and PI3K/Akt are unlikely to be involved in MSeA-induced activation of Nrf2 and the subsequent expression of NQO-1.

**Figure 6:**
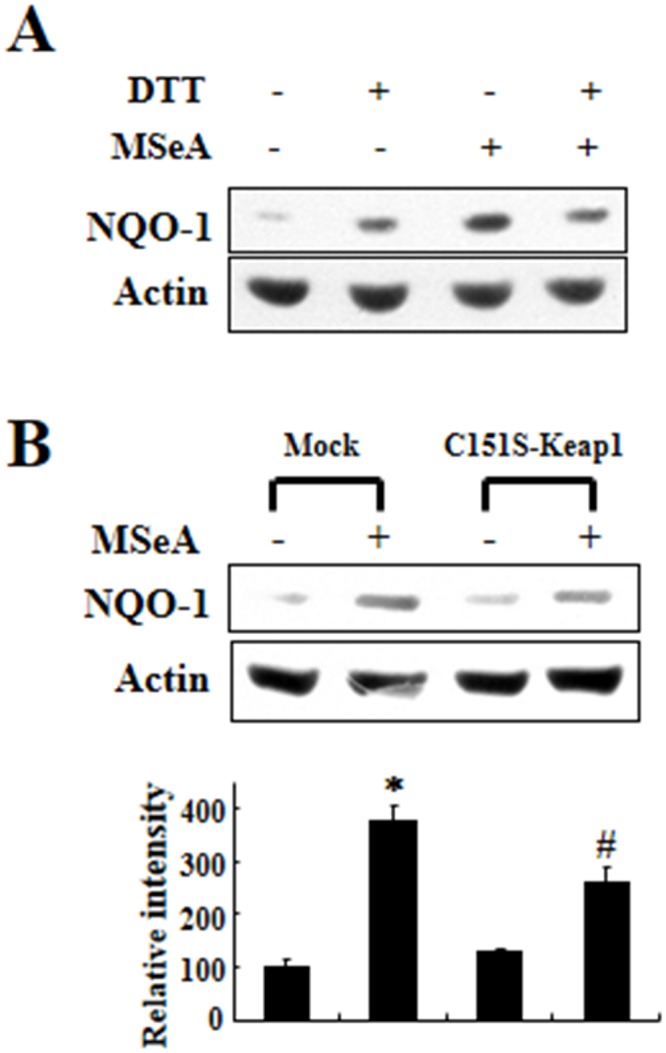
Effects of DTT and Keap1 cysteine 151 mutation on MSeA-induced NQO-1 expression **A.** Chang liver cells were preincubated for 1 h with 1 mM DTT followed by MSeA (3 μM) treatment for 24 h and harvested for measuring NQO-1 expression. **B.** An expression plasmid for Keap1 cysteine 151 mutant (C151S) and a mock vector were transfected into Chang liver cells. After 24-h transfection, the cells were treated with 3 μM MSeA for additional 24-h, and NQO-1 expression levels were determined by Western blot analysis (*P < 0.01 vs. mock alone, #*P* < 0.05 vs. mock plus MSeA).

The Keap1 protein contains a number of cysteine residues some of which can react with thiol-reactive chemicals [14]. Previous studies have demonstrated that specific cysteine residues in Keap1 are modified by electrophilic Nrf2 activators [18], which diminishes the interaction between Nrf2 and Keap1. To test the possible involvement of cysteine residues in Keap1 in the activation of Nrf2 following exposure to MSeA, we first examined the effect of the thiol-reducing agent dithiothreitol (DTT) on MSeA-induced NQO-1 expression. Pretreatment of Chang liver cells with DTT abrogated MSeA-induced NQO-1 upregulation (Figure 6). Of the 27 cysteine residues present in human Keap1, Cys-151 was reported to be the most reactive cysteine to undergo modification by some electrophilic Nrf2 activators [19, 20]. This prompted us to examine whether Cys-151 of Keap1 could be modified upon MSeA treatment in Chang liver cells. The MSeA-induced NQO-1 expression was partly inhibited in the cells transfected with the Keap1 mutant construct, in which Cys-151 was replaced by serine (Figure 6B). Taken together, these findings suggest that MSeA can activate Nrf2 signaling through direct interaction with Keap1, possibly at the Cys-151 residue.

### MSeA induces NQO-1 expression in mouse liver *in vivo*

**Figure 7:**
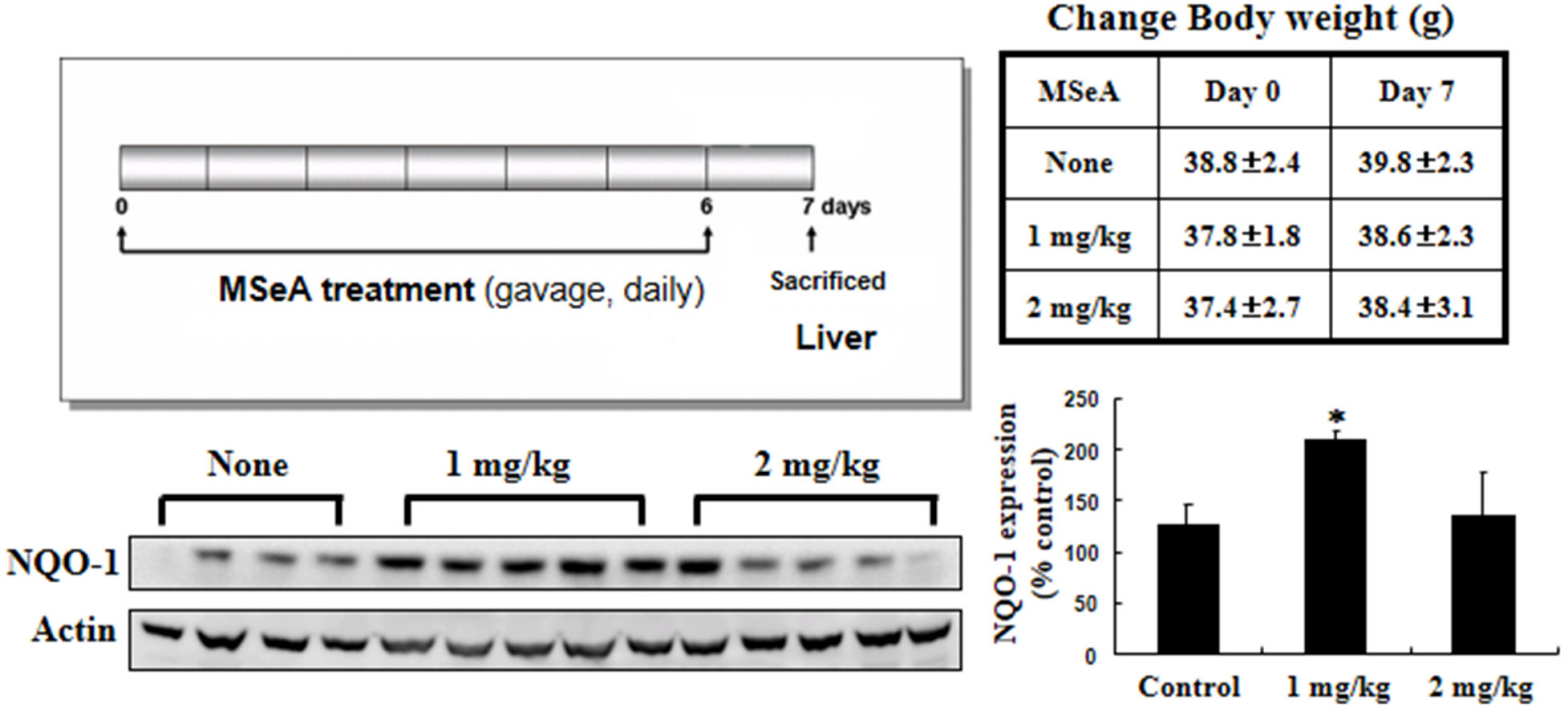
Effects of MSeA on NQO-1 expression in mouse liver Male ICR mice were treated with MSeA (1 or 2 mg/kg) by gavage for 7 days. Mice were killed by cervical dislocation, and liver tissues were collected for Western blot analysis to measure NQO-1 expression (*P < 0.01 vs. control ).

To confirm whether MSeA induces NQO-1 *in vivo*, we also examined the hepatic NQO-1 expression in mice after intragastric administration of MSeA (1 or 2 mg/mouse) once per a day for 7 days. Interestingly, administration of low dose (1 mg/kg) of MSeA reduced the hepatic NQO-1 expression while the higher dose (2 mg/kg) was ineffective (Figure 7).

### Methylselenol also enhances NQO-1 expression

**Figure 8:**
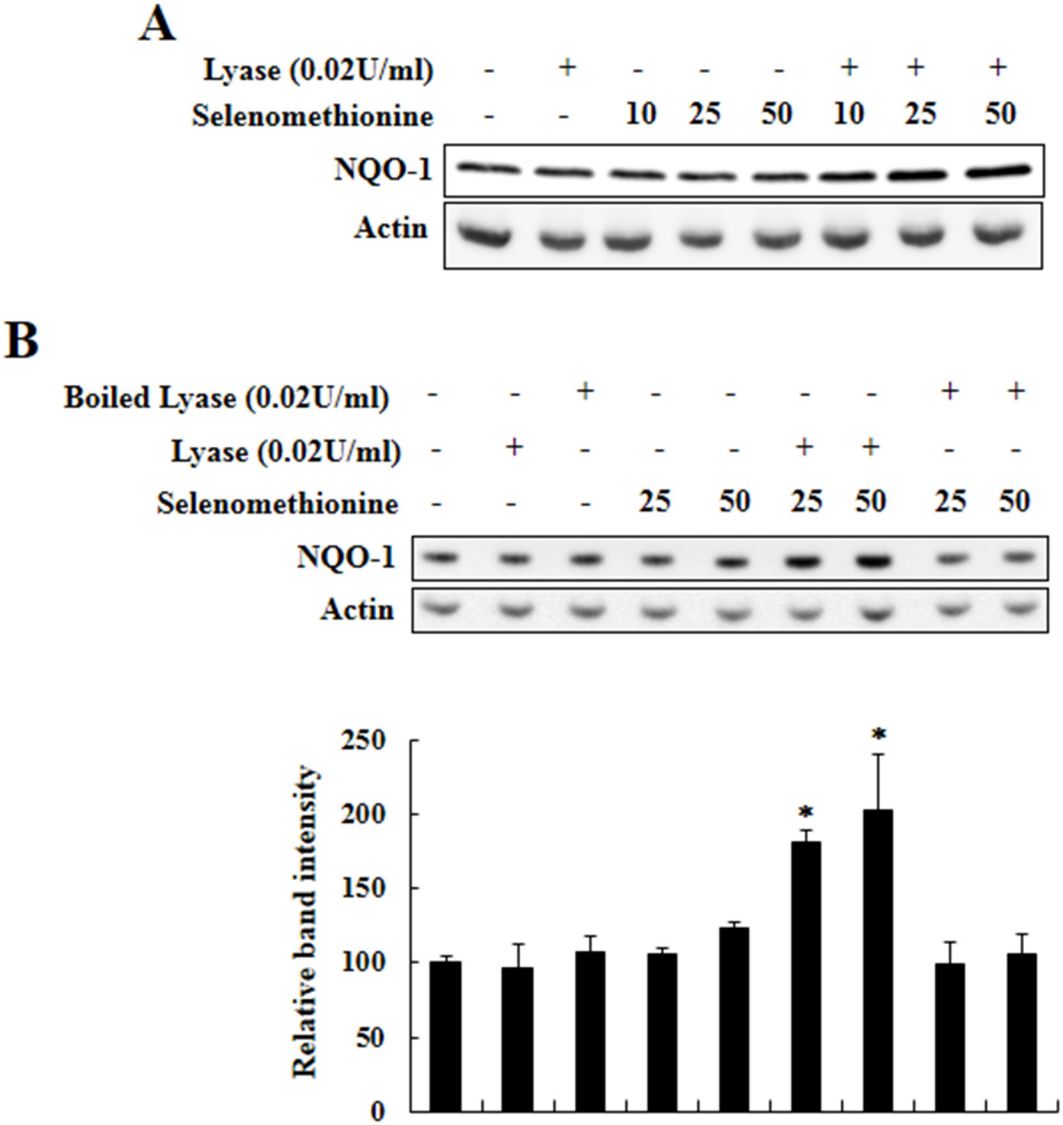
Induction of NQO-1 expression by methylselenol **A.** Chang liver cells were incubated with varying concentrations of selenomethionine (0, 10, 25, and 50 μM) and L-methionine-α-deamino-γ-mercaptomethane-lyase (0.02 U/ml) for 24 h and harvested for Western blot analysis to measure NQO-1 expression. **B.** Chang liver cells were incubated with varying concentrations of selenomethionine (0, 10, 25, 50 μM) and intact or boiled L-methionine-α-deamino-γ-mercaptomethane-lyase (0.02 U/ml) for 24-h, and NQO-1 expression was determined as above (*P < 0.01 vs. control).

MSeA is an immediate precursor of methylselenol. Thus, we determined whether the Nrf2-induced NQO-1 upregulation by MSeA is induced via production of methylselenol. Beside its formation from MSeA, methylselenol is known to be generated alternatively from selenomethionine in the presence of L-methionine-α-deamino-γ-mercaptomethane-lyase activity. As shown in Figure 8, incubation of Chang liver cells with both L-methionine-α-deamino-γ-mercaptomethane-lyase and selenomethionine increased NQO-1 expression, while incubation with either the enzyme or selenomethionine alone as well as boiled enzyme was inactive. These results imply the involvement of methylselenol in MSeA-induced NQO-1 expression.

## DISCUSSION

Selenium and its metabolites have been known to possess cancer chemopreventive potential, which is partly ascribed to their antioxidant properties. However, the detailed molecular mechanisms by which the selenium metabolites exert antioxidative and cancer chemopreventive effects remain still unanswered. Previously, Xiao *et al.* tested 27 Se compounds for their ability to induce carcinogen detoxifying enzyme activity in murine hepatoma (Hepa1c1c7) cells and identified dimethyl diselenide and MSeA as the two most potent inducers of NQO-1 [21]. Interestingly, dimethyl diselenide and MSeA are known to be readily reduced to methylselenol. In our study, we found that MSeA induced NQO-1 expression in both cultured human Chang liver cells and in mouse liver *in vivo*. Notably, selenomethionine plus L-methionine-α-deamino-γ-mercaptomethane-lyase which can directly generate methylselenol, also induced NQO-1 expression, whereas sodium selenite and selenomethionine alone, commonly used for clinical trials, were ineffective in inducing NQO-1 expression.

Depending on the type and the formulation, Se compounds have different mode of anticarcinogenic or chemopreventive effects [22-25]. For instance, while MSeA induces caspase-dependent apoptosis, apoptotic cell death caused by selenite is caspase-independent [22]. Selenite was more effective in inducing apoptosis than MSeA in LNCaP human prostate cancer cells, while MSeA exerted a more potent proapoptotic activity than did selenite in DU145 human prostate cancer cells [25]. Moreover, selenite reduced cell invasion via down-regulation of both MMP-2 and MMP-9, whereas MSeA-induced suppression of cell invasion was found to be mediated through inhibition of only MMP-2 [23, 24].

Two selenium metabolites are critical for chemoprevention. One is hydrogen selenide from inorganic selenium compounds, such as selenate and selenite; the other is methylselenol generated from organic selenium compounds, such as selenomethionine and Se-methylselenocysteine [24]. In the present study, we tested 3 major selenium metabolites defined according to the Se excretion pathways (Supplementary Figure 4); sodium selenite as a precursor of hydrogen selenide (H_2_Se), MSeA as an immediate precursor of methylselenol, and dimethylselenide (CH_3_SeCH_3_) as a methylation product of methyselenol. MSeA induced NQO-1 expression in Chang liver cells, but sodium selenite and dimethylselenide showed no remarkable effect. These results suggest that methylselenol plays a key role in antioxidant/carcinogen detoxifying enzyme induction among various selenium compounds. Production of methylselenol from sodium selenite, although possible *in vivo*, may not take place sufficiently enough to provoke NQO-1 inducing effects. Therefore, organoselenium products may have more potential as a cancer chemopreventive than inorganic selenium.

The transcription factor Nrf2 plays a master regulator of antioxidant and other cytoprotective gene expression. The mechanism responsible for Nrf2 activation is largely classified into two categories, that is, 1) modulation of protein kinase pathways and 2) modification of Keap1 cysteines, depending on the types of stimuli and cells [26-29]. In unstressed cells, Nrf2 located in the cytoplasm is rapidly degraded through polyubiquitination by the Keap1-Cul3 E3 ubiquitin ligase complex [30-32]. When cells are challenged with oxidative or electrophilic stress, Nrf2 evades polyubiquitination by the Keap1-Cul3 E3 ubiquitin ligase complex [33]. Recently, Liu *et al.* also reported that MSeA significantly down-regulated Keap1, induced nuclear accumulation of Nrf2 and enhanced ARE-promoter activity in human oesophageal squamous carcinoma cells [34]. In our present study, MSeA did not influence the mRNA expression of Keap1 (data not shown), but reduced the steady-state level of Keap1 protein.

The Keap1 protein of the untreated cells is detected as a 70 kDa monomer, whereas the Keap1 protein in cells stimulated with Nrf2 activators comprises a series of high molecular weight (HMW) bands with a molecular mass of greater than 150 kDa [14, 35]. Keap1 brings Nrf2 into close proximity to Cul3 ubiquitin ligase, rendering Nrf2 subjected to ubiquitination and degradation. However, adduction of Keap1 by electrophiles may trigger a switching of Cul3-dependent ubiquitination from Nrf2 to Keap1, leading to Nrf2 activation [36]. The formation of HMW Keap1 upon treatment with some electrophiles and chemopreventive agents, such as sulforaphane, ebselen, and quercetin, was reported [14, 35-38]. Furthermore, it has been proposed that electrophiles induce dimerization of Keap1 monomers via disulfide linkage between via Cys-273 and Cys-288 residues [39].

**Figure 9:**
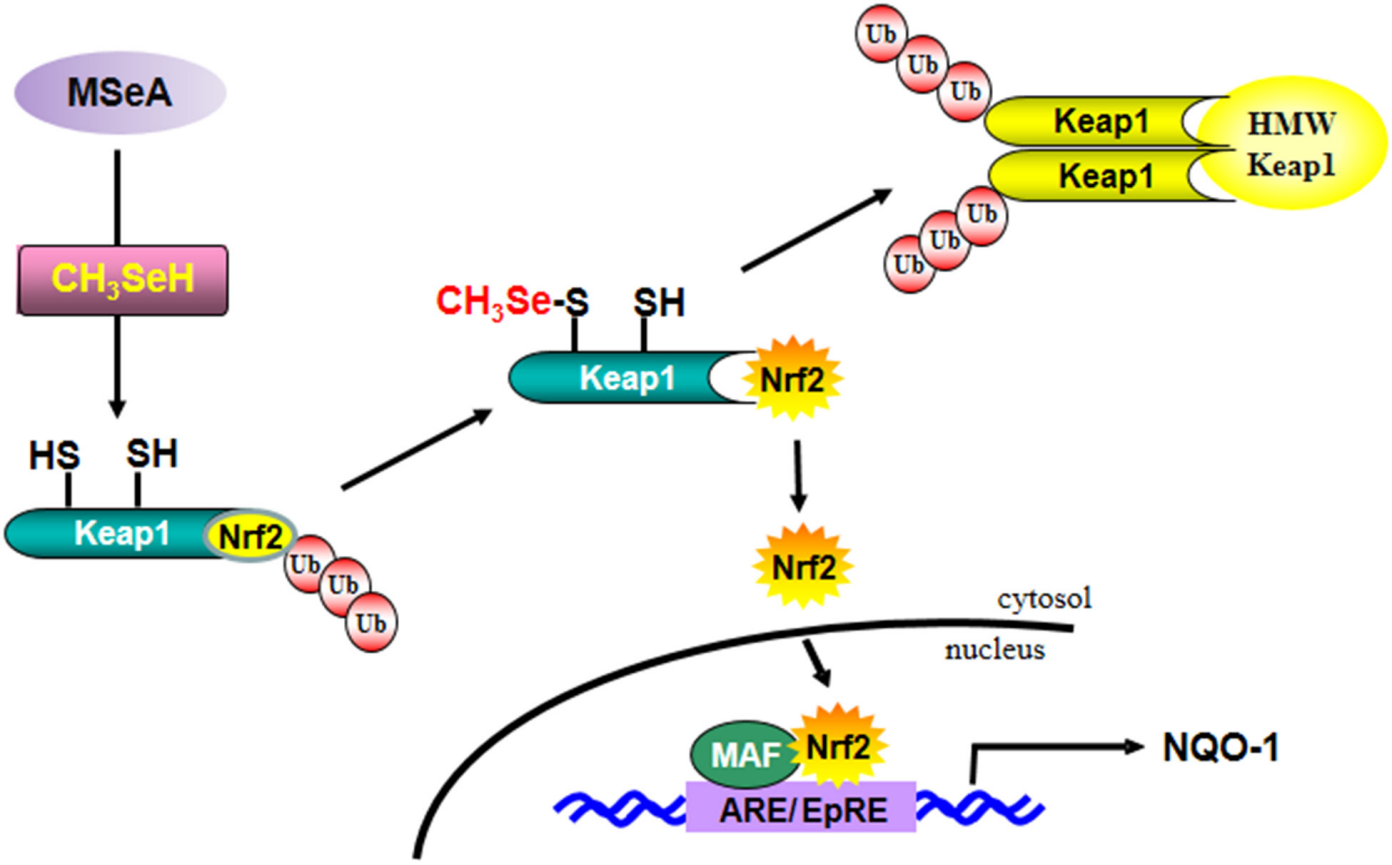
A proposed pathway for MSeA-induced Nrf2 activation and NQO-1 expression Abbreviations: MSeA, methylseleninic acid; CH_3_SeH, methylselenol; HWP Keap1, high molecular weight Keap1; ARE, antioxidant response elements; EpRE, electrophile response elements.

Human Keap1 has 27 highly reactive cysteine residues. Among these, Cys-151, Cys-273, and Cys-288 were demonstrated to be essential for regulating Nrf2 degradation [14, 40]. Especially, Cys-151 was highly and consistently modified by some chemopreventive and chemoprotective phytochemicals [26, 41, 42]. Brigelius-Flohe has suggested that selenium metabolites, such as selenols or selenenic acids, can modify protein thiols to form selenotrisulfides (S-Se-S), selenylsulfides (Se-S), protein disulfides (S-S), or diselenides (Se-Se) [43]. In this study, we found that the thiol reducing agent DTT could attenuate the activity of MSeA at least by keeping Keap1 thiol in a reduced state, thereby hampering interaction between MSeA and a critical Keap1 cysteine. These effects were abated in cells harboring the Keap1 C151S mutant construct, indicative of the Cys-151 residue of Keap1 as a putative target of MSeA in its inducing Nrf2-mediated NQO-1 expression. We speculate that MSeA-induced modifications of Keap1 thiols including Cys-151 may trigger the Keap1 ubiquitination and thereafter proteasomal degradation, while limiting the Nrf2 access to the Cul3-dependent ubiquitin ligase complex. This will stabilize Nrf2 and facilitate its dissociation from Keap1 for nuclear translocation and ARE binding (Figure 9).

Primarily, Nrf2 protects cells from oncogenic insults, including ROS and electrophilic carcinogenic species. However, once malignant transformation has occurred within a cell, Nrf2-Keap1 signaling can be hijacked by transformed cells and functions to protect the tumor from oxidative stress and chemo- or radiotherapy-induced cytotoxicity [44]. Thus, Nrf2 plays dual roles in cancer prevention and progression depending on the cellular microenvironment, including redox status. Though the constitutively activated Nrf2 and subsequent overexpression of antioxidant and related stress response proteins can confer survival advantage and resistance to anticancer therapy in cancer cells, this does not necessarily exclude the benefit of Nrf2 inducers in normal cells. As the Nrf2 is already overactivated in tumors, the additional effects by Nrf2 activators, if any, would be marginal. Under conventional chemo- or radiation-therapy, normal cells as well as cancer cells are under oxidative stress. In this context, Nrf2 induction by chemopreventive agents, such as MSeA that prevents normal cells from oxidative damage which often arises from anticancer therapy is considered to be beneficial in cancer patients.

Intragastric administration of MSeA induced the expression of NQO-1 in mouse liver. Interestingly, administration of low dose (1 mg/kg) of MSeA was more effective than higher dose (2 mg/kg) in inducing NQO-1 expression (Figure 7). Several dietary components, including selenium, have been shown to have a typical hormetic dose response [45, 46]. Likewise, MSeA may have hormetic effects that are achieved through alterations in expression of stress responsive genes involved in various antioxidant defense and repair pathways.

In summary, MSeA, as a precursor of methylselenol, activates Nrf2 and subsequently induces expression of NQO-1 by directly modifying Keap1 (Figure 9). These findings provide a novel mechanism by which MSeA, among selenium metabolites, exhibits remarkable capability in inducing antioxidant/stress responsive gene expression.

## MATERIALS AND METHODS

### Cell culture

Chang liver cells were kindly supplied by Dr. Kyu-Won Kim (College of Pharmacy, Seoul National University, Seoul, South Korea). The cells were suspended in DMEM (Gibco/Invitrogen, Carlsbad, CA) medium supplemented with 50 mg/l gentamicin (Life Technologies, Inc., Rockville, MD) and 10% heat-inactivated fetal bovine serum (FBS; Gibco/Invitrogen, Carlsbad, CA) and maintained at 37°C in a humidified atmosphere composed of 5% CO_2_/95% air.

### NQO-1 activity assay

The principle of the NQO-1 assay is that glucose 6-phosphate and glucose-6-phosphate dehydrogenase continually generate NADPH, which is used by quinone reductase to transfer electrons to menadione. The resulting menadiol reduces MTT to form blue formazan, which can be measured with an ELISA plate reader at 620 nm [47]. After treatment with MSeA, cells (4 × 10^3^ cells/100 μl in 96-well plate) were washed with phosphate buffered saline (PBS) and lysed by incubation at 37°C for 10 min with 50 μl of a solution containing 0.8% digitonin. The plates were then agitated on an orbital shaker for an additional 10 min at 25°C. Menadione (1 μl of 50 mM stock solution dissolved in acetonitrile per milliliter of reaction mixture) was added just before the reaction mixture was dispensed into the microtiter plates. Absorbance at 620 nm was then made at 5-min intervals. NQO-1 activity was determined by calculation of the difference in slope between samples and baseline (extinction coefficient, 11,300 M/cm at 620 nm).

### Immunofluorescence staining

Chang liver cells placed on four-well chamber slides were treated with MSeA for 6 h. Cells were rinsed rapidly with PBS and then fixed for 30 min at room temperature with 4% formaldehyde. After washing with PBS, the fixed cells were incubated further for 2 h at room temperature in PBS containing 10% bovine serum albumin and 0.5% Tween-20. The nuclear translocation of Nrf2 was visualized using a rabbit polyclonal antibody. The Nrf2 antibody was added after 1:100 dilution with the blocking buffer, and cells were incubated overnight at 4°C. Afterwards, the incubated cells were washed with PBS and then labeled with diluted (1:1000) FITC-conjugated goat anti-rabbit IgG (Zymed Laboratories) and incubated for additional 1 h at room temperature. Cells were then rinsed with PBS and stained with PI for 10 min. After washing with PBS, cells were analyzed under a confocal microscope and photographed (Leica Microsystems Heidelberg GmbH).

### Nuclear protein extraction and the electrophoretic mobility gel shift assay (EMSA)

After treatment with MSeA, cells were washed with PBS, centrifuged and suspended in the ice-cold isotonic buffer A [a plasma membrane lysis buffer containing 10 mM HEPES (pH 7.9), 1.5 mM MgCl_2_, 10 mM KCl, 0.5 mM DTT, and 0.2 mM phenylmethylsulfonyl fluoride (PMSF)]. Following incubation on ice for 10 min, the lysate was centrifuged, and the resulting pellets were resuspended in ice-cold buffer C [a nuclear membrane lysis buffer containing 20 mM HEPES (pH 7.9), 20% glycerol, 420 mM NaCl, 1.5 mM MgCl_2_, 0.2 mM EDTA, 0.5 mM DTT, and 0.2 mM PMSF] and incubated for 20 min on ice. After a vigorous vortex-mixing, the lysed nuclear fraction was centrifuged, and the supernatant was collected and stored at -70°C for EMSA. The oligonucleotide containing the consensus sequence that binds to Nrf2 was labeled with [γ-^32^P]ATP using T4 polynucleotide kinase and separated from the unincorporated [γ-^32^P]ATP by gel filtration using a nick spin column (Pharmacia). After mixing the radiolabeled Nrf2 oligonucleotide (100,000 cpm) with 10 μg of the nuclear extract in gel shift binding buffer [4% glycerol, 1 mM EDTA, 1 mM DTT, 100 mM NaCl, 10 mM Tris–HCl (pH 7.5), and 0.1 mg/ml sonicated salmon sperm DNA], the mixture was kept on ice for 15 min. The DNA–protein complexes formed were resolved by 6% non-denaturating polyacrylamide gel electrophoresis carried out at 170 V for 2 h. After a vacuum-drying, the gel was exposed to X-ray film and kept at -70°C for autoradiography.

### Immunoprecipitation and Western blot analysis

After treatment with MSeA, Chang liver cells (3 × 10^6^) were lysed with modified RIPA buffer containing 10 mM Tris-HCl (pH 8.0), 150 mM NaCl, 1 mM EDTA, 0.1% Nonidet P-40, 1% deoxycholate, 50 mM sodium fluoride, 50 mM sodium orthovanadate, 1 mM PMSF, and proteinase inhibitor cocktail (Nacarai Tesque, Kyoto, Japan). The lysates were homogenized in an ultrasonicator for 10 sec twice and incubated on ice for 30 min. The homogenates were centrifuged at 14,000 *g* for 15 min at 4°C. The supernatants were collected, and the protein concentration was determined by the protein assay kit (Bio-Rad Laboratories). For immunoprecipitation, whole-cell lysates containing 1 mg of proteins were precleared with protein A-Sepharose beads (Amersham Pharmacia Biotech) for 1 h and incubated with 2 μg of anti-Nrf2 or anti-Keap1 antibody for 4 h. Immunoprecipitated complexes were washed five times with RIPA buffer and then boiled in SDS sample buffer for 5 min. Either the immunoprecipitation products or the whole-cell lysates containing 40 μg of proteins were run on 8% SDS-PAGE and electrophoretically transferred to PVDF membrane (Amersham Pharmacia Biotech). After blotting, the membrane was incubated with specific antibody overnight at 4°C and further incubated for 1 h with HRP-conjugated secondary antibody. Bound antibodies were detected using the ECL system and the relative amounts of proteins associated with specific antibody were quantified using Lumi Vision Imager soft ware (TAITEC).

### Chromatin immunoprecipitation (ChIP) assay

Chang liver cells were grown in 100-mm dishes for 24 h and treated with MSeA for another 6 h. The ChIP assay was performed with the ChIP-IT kit (Active Motif, Carlsbad, CA, USA) according to the manufacturer's instructions. Briefly, cellular proteins and DNA were cross-linked with 1% formaldehyde in DMEM for 20 min at room temperature. The lysates were sonicated for 20 cycles (20-s pulse and 30-s rest on ice) and the isolated chromatin was run on a 2% agarose gel to check for shearing efficiency. Subsequently, Nrf2 bound to chromatin complexes were immunoprecipitated with anti-Nrf2 antibody. Rabbit IgG was used as control to check the specificity. PCR was performed with the eluted genomic DNA using human NQO-1-ARE primers (forward, 5′-CAGGCCACTTGAAGAGAGAG-3′; reverse, 5′-AGTGAAACCGAAACGGAGC-3′). Ten percent of the chromatin DNA used for immunoprecipitation was also subjected to PCR analysis and indicated as input.

### Animal study

Fifteen 6-week-old male ICR mice were purchased from Orient Bio (Sungnam, South Korea) and grouped into three (*n* = 5 per treatment group). MSeA (1 or 2 mg/kg/day) was mixed in tap water and given by gavage for 7 days. After treatment with MSeA for 7 days, mouse livers were perfused with PBS. The liver tissues were then taken out, flash-frozen in lipid nitrogen, and kept at -70°C for subsequent analysis.

### Statistical evaluation

Values were expressed as the mean ± SD of the results obtained from at least three independent experiments. Statistical significance of the obtained data was determined by conducting Student’s *t*-test, and a *p*-value of less than 0.01 was considered to be statistically significant.

### Supplementary information

Detailed experimental procedures for Reagents, RT-PCR, Western blot, and transient transfection and the luciferase reporter assay can be found in *Supplementary information* Text.

## SUPPLEMENTARY MATERIALS FIGURES



## References

[1] Stadtman TC (1996). Selenocysteine. Annu Rev Biochem.

[2] Clark LC, Combs GF, Turnbull BW, Slate EH, Chalker DK, Chow J, Davis LS, Glover RA, Graham GF, Gross EG, Krongrad A, Lesher JL, Park HK (1996). Effects of selenium supplementation for cancer prevention in patients with carcinoma of the skin. A randomized controlled trial. Nutritional Prevention of Cancer Study Group. JAMA.

[3] Rayman MP (2012). Selenium and human health. Lancet.

[4] Nicastro HL, Dunn BK (2013). Selenium and prostate cancer prevention: insights from the selenium and vitamin E cancer prevention trial (SELECT). Nutrients.

[5] Marshall JR, Tangen CM, Sakr WA, Wood DP, Berry DL, Klein EA, Lippman SM, Parnes HL, Alberts DS, Jarrard DF, Lee WR, Gaziano JM, Crawford ED (2011). Phase III trial of selenium to prevent prostate cancer in men with high-grade prostatic intraepithelial neoplasia: SWOG S9917. Cancer Prev Res (Phila).

[6] Weekley CM, Aitken JB, Finney L, Vogt S, Witting PK, Harris HH (2013). Selenium metabolism in cancer cells: the combined application of XAS and XFM techniques to the problem of selenium speciation in biological systems. Nutrients.

[7] Yang L, Pascal M, Wu XH (2013). Review of selenium and prostate cancer prevention. Asian Pac J Cancer Prev.

[8] Chen YC, Prabhu KS, Mastro AM (2013). Is selenium a potential treatment for cancer metastasis?. Nutrients.

[9] Pinto JT, Lee JI, Sinha R, MacEwan ME, Cooper AJ (2011). Chemopreventive mechanisms of α-keto acid metabolites of naturally occurring organoselenium compounds. Amino Acids.

[10] Lunoe K, Gabel-Jensen C, Sturup S, Andresen L, Skov S, Gammelgaard B (2011). Investigation of the selenium metabolism in cancer cell lines. Metallomics.

[11] Ip C, Thompson HJ, Zhu Z, Ganther HE (2000). In vitro and in vivo studies of methylseleninic acid: evidence that a monomethylated selenium metabolite is critical for cancer chemoprevention. Cancer Res.

[12] Jaiswal AK (2000). Regulation of genes encoding NAD(P)H:quinone oxidoreductases. Free Radic Biol Med.

[13] Dinkova-Kostova AT, Talalay P (2010). NAD(P)H:quinone acceptor oxidoreductase 1 (NQO1), a multifunctional antioxidant enzyme and exceptionally versatile cytoprotector. Arch Biochem Biophys.

[14] Zhang DD, Hannink M (2003). Distinct cysteine residues in Keap1 are required for Keap1-dependent ubiquitination of Nrf2 and for stabilization of Nrf2 by chemopreventive agents and oxidative stress. Mol Cell Biol.

[15] Hayes JD, McMahon M (2001). Molecular basis for the contribution of the antioxidant responsive element to cancer chemoprevention. Cancer Lett.

[16] Itoh K, Wakabayashi N, Katoh Y, Ishii T, Igarashi K, Engel JD, Yamamoto M (1999). Keap1 represses nuclear activation of antioxidant responsive elements by Nrf2 through binding to the amino-terminal Neh2 domain. Genes Dev.

[17] Surh YJ, Kundu JK, Na HK (2008). Nrf2 as a master redox switch in turning on the cellular signaling involved in the induction of cytoprotective genes by some chemopreventive phytochemicals. Planta Med.

[18] Dinkova-Kostova AT, Holtzclaw WD, Cole RN, Itoh K, Wakabayashi N, Katoh Y, Yamamoto M, Talalay P (2002). Direct evidence that sulfhydryl groups of Keap1 are the sensors regulating induction of phase 2 enzymes that protect against carcinogens and oxidants. Proc Natl Acad Sci U S A.

[19] Sekhar KR, Rachakonda G, Freeman ML (2010). Cysteine-based regulation of the CUL3 adaptor protein Keap1. Toxicol Appl Pharmacol.

[20] Yamamoto T, Suzuki T, Kobayashi A, Wakabayashi J, Maher J, Motohashi H, Yamamoto M (2008). Physiological significance of reactive cysteine residues of Keap1 in determining Nrf2 activity. Mol Cell Biol.

[21] Xiao H, Parkin KL (2006). Induction of phase II enzyme activity by various selenium compounds. Nutr Cancer.

[22] Jiang C, Ganther H, Lu J (2000). Monomethyl selenium--specific inhibition of MMP-2 and VEGF expression: implications for angiogenic switch regulation. Mol Carcinog.

[23] Yoon SO, Kim MM, Chung AS (2001). Inhibitory effect of selenite on invasion of HT1080 tumor cells. J Biol Chem.

[24] Park JM, Kim A, Oh JH, Chung AS (2007). Methylseleninic acid inhibits PMA-stimulated pro-MMP-2 activation mediated by MT1-MMP expression and further tumor invasion through suppression of NF-KB activation. Carcinogenesis.

[25] Li GX, Hu H, Jiang C, Schuster T, Lu J (2007). Differential involvement of reactive oxygen species in apoptosis induced by two classes of selenium compounds in human prostate cancer cells. Int J Cancer.

[26] Eggler AL, Small E, Hannink M, Mesecar AD (2009). Cul3-mediated Nrf2 ubiquitination and antioxidant response element (ARE) activation are dependent on the partial molar volume at position 151 of Keap1. Biochem J.

[27] Kong AN, Owuor E, Yu R, Hebbar V, Chen C, Hu R, Mandlekar S (2001). Induction of xenobiotic enzymes by the MAP kinase pathway and the antioxidant or electrophile response element (ARE/EpRE). Drug Metab Rev.

[28] Huang HC, Nguyen T, Pickett CB (2002). Phosphorylation of Nrf2 at Ser-40 by protein kinase C regulates antioxidant response element-mediated transcription. J Biol Chem.

[29] Martin D, Rojo AI, Salinas M, Diaz R, Gallardo G, Alam J, De Galarreta CM, Cuadrado A (2004). Regulation of heme oxygenase-1 expression through the phosphatidylinositol 3-kinase/Akt pathway and the Nrf2 transcription factor in response to the antioxidant phytochemical carnosol. J Biol Chem.

[30] Dhakshinamoorthy S, Jaiswal AK (2001). Functional characterization and role of INrf2 in antioxidant response element-mediated expression and antioxidant induction of NAD(P)H:quinone oxidoreductase1 gene. Oncogene.

[31] McMahon M, Itoh K, Yamamoto M, Hayes JD (2003). Keap1-dependent proteasomal degradation of transcription factor Nrf2 contributes to the negative regulation of antioxidant response element-driven gene expression. J Biol Chem.

[32] Zhang DD, Lo SC, Cross JV, Templeton DJ, Hannink M (2004). Keap1 is a redox-regulated substrate adaptor protein for a Cul3-dependent ubiquitin ligase complex. Mol Cell Biol.

[33] Motohashi H, Yamamoto M (2004). Nrf2-Keap1 defines a physiologically important stress response mechanism. Trends Mol Med.

[34] Liu M, Hu C, Xu Q, Chen L, Ma K, Xu N, Zhu H (2015). Methylseleninic acid activates Keap1/Nrf2 pathway via up-regulating miR-200a in human oesophageal squamous cell carcinoma cells. Biosci Rep.

[35] Hong F, Sekhar KR, Freeman ML, Liebler DC (2005). Specific patterns of electrophile adduction trigger Keap1 ubiquitination and Nrf2 activation. J Biol Chem.

[36] Hong F, Freeman ML, Liebler DC (2005). Identification of sensor cysteines in human Keap1 modified by the cancer chemopreventive agent sulforaphane. Chem Res Toxicol.

[37] Sakurai T, Kanayama M, Shibata T, Itoh K, Kobayashi A, Yamamoto M, Uchida K (2006). Ebselen, a seleno-organic antioxidant, as an electrophile. Chem Res Toxicol.

[38] Tanigawa S, Fujii M, Hou DX (2007). Action of Nrf2 and Keap1 in ARE-mediated NQO1 expression by quercetin. Free Radic Biol Med.

[39] Wakabayashi N, Dinkova-Kostova AT, Holtzclaw WD, Kang MI, Kobayashi A, Yamamoto M, Kensler TW, Talalay P (2004). Protection against electrophile and oxidant stress by induction of the phase 2 response: fate of cysteines of the Keap1 sensor modified by inducers. Proc Natl Acad Sci U S A.

[40] Kobayashi M, Li L, Iwamoto N, Nakajima-Takagi Y, Kaneko H, Nakayama Y, Eguchi M, Wada Y, Kumagai Y, Yamamoto M (2009). The antioxidant defense system Keap1-Nrf2 comprises a multiple sensing mechanism for responding to a wide range of chemical compounds. Mol Cell Biol.

[41] Eggler AL, Luo Y, van Breemen RB, Mesecar AD (2007). Identification of the highly reactive cysteine 151 in the chemopreventive agent-sensor Keap1 protein is method-dependent. Chem Res Toxicol.

[42] Luo Y, Eggler AL, Liu D, Liu G, Mesecar AD, van Breemen RB (2007). Sites of alkylation of human Keap1 by natural chemoprevention agents. J Am Soc Mass Spectrom.

[43] Brigelius-Flohe R (2008). Selenium compounds and selenoproteins in cancer. Chem Biodivers.

[44] Abdul-Aziz A, MacEwan DJ, Bowles KM, Rushworth SA (2015). Oxidative stress responses and NRF2 in human leukaemia. Oxid Med Cell Longev.

[45] Mattson MP (2008). Dietary factors, hormesis and health. Ageing Res Rev.

[46] Hayes DP (2007). Nutritional hormesis. Eur J Clin Nutr.

[47] Lee YS, Kang YS, Lee JS, Nicolova S, Kim JA (2004). Involvement of NADPH oxidase-mediated generation of reactive oxygen species in the apototic cell death by capsaicin in HepG2 human hepatoma cells. Free Radic Res.

