# Supplementary material for: Methylseleninic acid induces NAD(P)H:quinone oxidoreductase-1 expression through activation of NF-E2-related factor 2 in Chang liver cells
Source: Oncotarget. 2016 Jun 25;9(3):3014–28. doi: 10.18632/oncotarget.10289 (PMC5790442; doi:10.18632/oncotarget.10289)
Supplement: Supplementary file 1 [file oncotarget-09-3014-s001.pdf]

SUPPLEMENTARY DATA

Reagents

MSeA was provided by Dr. Julian Spallholz of PharmaSe, Inc. (Lubbock, TX). L-Methionine γ-lyase was purchased from Wako Pure Chemical Industries, Ltd. (Osaka, Japan). MTT, NAC, FAD, NADP, NADPH, glucose-6-phosphate, glucose-6-phosphate dehydrogenase, 2-methyl-1,4-naphthoquinone (menadione), digitonin, and 3,3'-methylenebis(4-hydroxycoumarin) (dicumarol) were purchased from Sigma Chemical Co. (St. Louis, MO). Dulbecco's modified Eagle's medium (DMEM) and fetal bovine serum (FBS) were purchased from Gibco BRL (Grand Island, NY). An antibody against ubiquitin was purchased from Cell Signaling Technology (Beverly, MA). Antibodies against Nrf2, Keap-1, NQO-1, and actin were products of Santa Cruz Biotechnology, Inc. (Santa Cruz, CA). Secondary antibodies for Western blotting were obtained from Zymed Laboratories Inc. (San Francisco, CA). MG132 was purchased from Calbiochem (San Diago, CA). pEF (control vector), pEF-dominant-negative Nrf2 (DN-Nrf2), and reporter gene-fusion constructs for luciferase (pTi-luciferase), wild-type ARE, and GC mutant ARE were kindly provided by Dr. Jeffery A. Johnson (University of Wisconsin–Madison, Madison, WI). The enhanced chemiluminescence (ECL) detection kit and [γ-^{32}P]ATP were purchased from Amersham Pharmacia Biotech (Buckinghamshire, UK). The oligonucleotide containing the Nrf2 binding sequence and the luciferase assay kit with reporter lysis buffer were purchased from Promega (Madison, WI).

Reverse transcriptase–polymerase chain reaction (RT-PCR)

Total RNA was isolated from Chang liver cells using TRIzol® (GibcoBRL, Grand Island, NY) by following the manufacturer's instructions. RT-PCR primers used in this study were as follows: (forward and reverse, respectively): NQO-1, 5'-CACACTCCAGCAGACGCCCG-3' and 5'-TGCCCAAGTGATGGCCCACAG-3', 555 bp; GAPDH, 5'-AAGGTCGGAGTCAACGGATT-3' and 5'-GCAGTGGGTCTCTCTCCT-3', 1,054 bp. To amplify the cDNA specific for NQO-1, 25 cycles of 95°C for 1 min, 60°C for 1 min, and 72°C for 1 min were carried out, and to amplify the housekeeping gene glyceraldehyde-3-phosphate dehydrogenase (GAPDH) to be used as an internal control, 26 cycles of 94°C for 1 min, 56°C for 2 min, and 72°C for 2 min were carried out. These amplification cycles were followed by a final extension at 72°C for 10 min. Amplification products were separated using 1.2% agarose gel electrophoresis, stained with ethidium bromide, and photographed under UV light. All primers were purchased from Bionics (Seoul, South Korea).

Western blot analysis

After treatment with MSeA, cells were washed with PBS and then lysed at 4°C for 30 min in the lysis buffer (150 mM NaCl, 50 mM Tris-HCl, pH 7.4, 25 mM NaF, 20 mM EGTA, 0.5% Triton X-100, 1 mM DTT and 1 mM Na_3VO_4 with protease inhibitor cocktail tablet). Nuclei and unlysed cellular debris were removed by centrifugation at 14,000 x *g* for 15 min at 4°C. The protein concentration in the cytosolic fraction was determined by using the BCA protein assay kit (Pierce Biotechnology, Inc.). Protein samples were separated on SDS polyacrylamide gel electrophoresis, and the separated proteins were transferred to polyvinylidene difluoride (PVDF) membrane at 300 mA for 4 h. To prevent the interaction between the antibodies and non-specific proteins, the PVDF membrane was blocked for 1 h at room temperature by immersion in fresh blocking buffer (0.1% Tween-20 in PBS, pH 7.4, containing 5% nonfat dry milk). Dilutions of primary antibodies were made in PBS containing 3% nonfat dry milk. The PVDF membrane blots were then incubated overnight at 4°C in a solution containing diluted primary antibodies. Following 3 washes with PBST (PBS containing 0.1% Tween-20), the blots were incubated further for 1 h at room temperature in a dish containing the horseradish peroxidase-conjugated secondary antibody dissolved in PBS containing 3% nonfat dry milk. Thereafter, the blots were washed again three times in PBST buffer, incubated with ECL solution (Amersham Pharmacia Biotech, Inc.) for 1 min and then visualized using a radiographic film according to the manufacturer's instructions.

Transient transfection and the luciferase reporter assay

Chang liver cells were seeded at a density of 2 × 10^5/well in a 6-well dish and grown to 60–70% confluence in the complete growth media. The cells in each well were co-transfected with 2 µg of the luciferase reporter plasmid construct harboring the Nrf2 binding site (pGL2-Nrf2) or the pCMV-β-galactosidase control vector using the WelFect-M™GOLD transfection reagent (WelGENE) according to the instructions supplied by the manufacturer. After an 18-h transfection, the medium was changed, and the cells were treated with MSeA for 6 h. The cells were then washed with PBS, lysed in 1 × reporter lysis buffer

(Promega). The lysed cell extract (20 µl) was mixed with 100 µl of the luciferase assay reagent, and the luciferase activity was determined using a luminometer (AutoLumat LB 953, EG&G Berthold). To normalize the luciferase activity, the *β*-galactosidase assay was conducted according to the supplier's instructions (Promega *β*-galactosidase assay enzyme assay system).

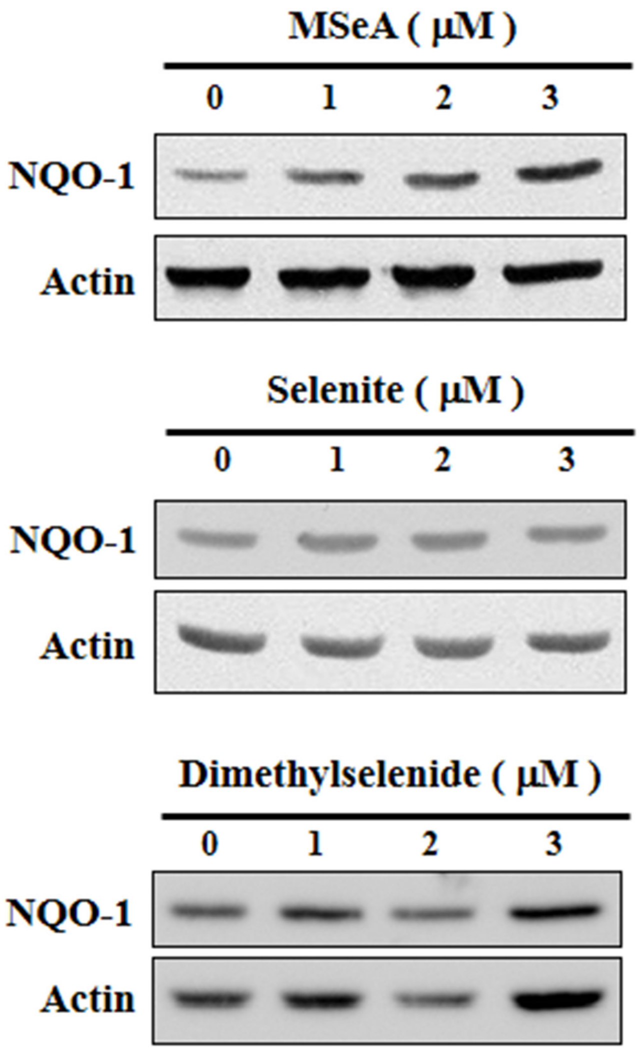

Supplementary Figure S1: Induction of NQO-1 expression by some representative selenium metabolites. Chang liver cells were incubated with indicated concentrations of MSeA, selenite and dimethylselenide (0, 1, 2, and 3 μM) for 24 h and harvested for measuring NQO-1 expression by Western blot analysis.

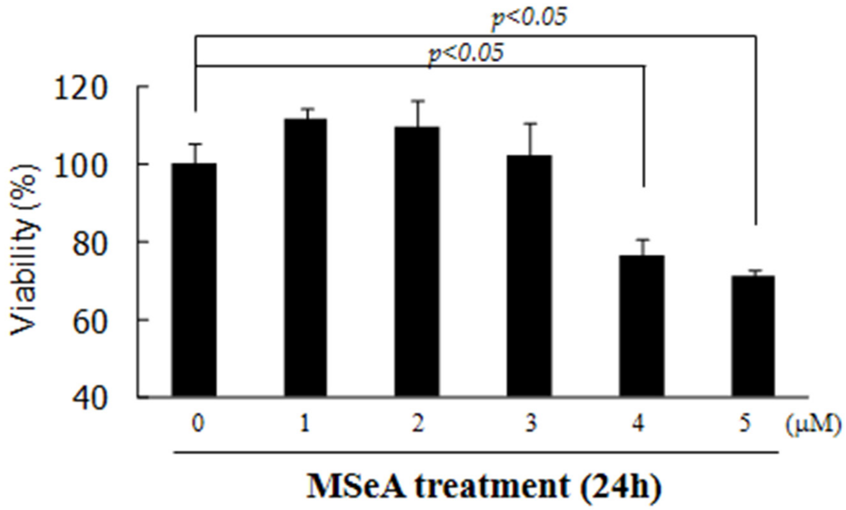

Supplementary Figure S2: Effects of MSeA on viability of Chang liver cells. The cells were treated with MSeA (0, 1, 2, 3, 4, and 5 μM) for 24 h. Cell viability was determined by the MTT assay. The data represent mean ± SD (n=3). Significant differences between the compared groups are indicated.

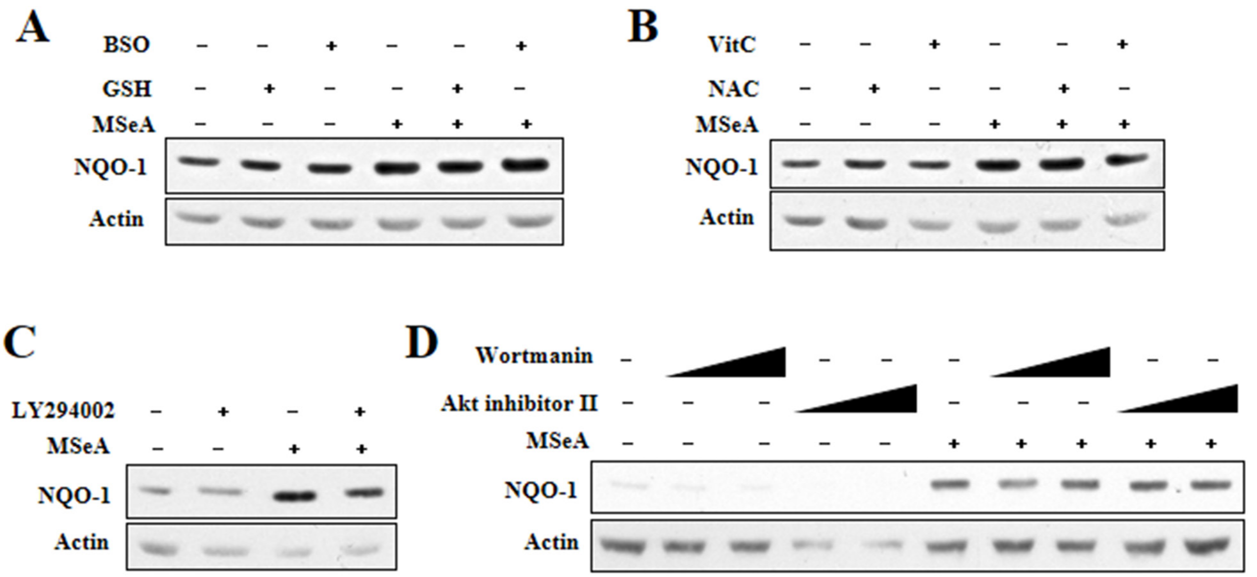

Supplementary Figure S3: Effects of redox modulators and inhibitors of PI3K-Akt signaling on MSeA-induced NQO-1 expression. Chang liver cells were preincubated for 1 h with 50 μM BSO or 5 mM GSH **A.** with 10 mM Vit. C or 5 mM NAC **B.** with 25 μM LY294002 **C.** or with 20 and 200 μM Wortmanin or 10 and 50 μM Akt inhibitor II **D.** followed by MSeA (3 μM) treatment for additional 24 h and harvested for Western blot analysis to measure NQO-1 expression.

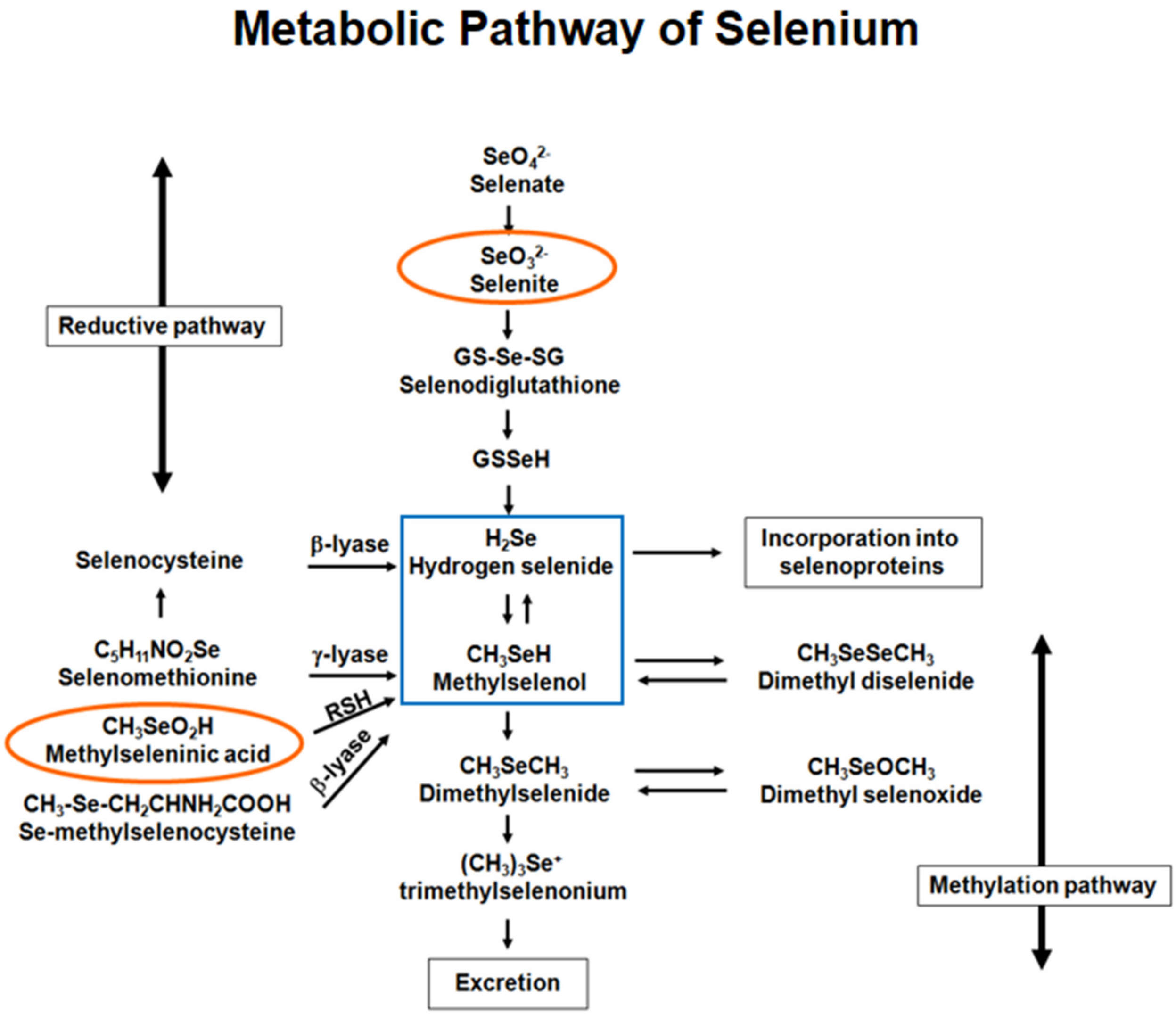

Supplementary Figure S4: A metabolic pathway of selenium.
